# Prioritising genetic testing for kidney stone disease in adults using a clinical scoring system

**DOI:** 10.1093/ckj/sfag008

**Published:** 2026-01-14

**Authors:** Federico Maffei Faccioli, Mario Gennaro Cozzolino, Shabbir H Moochhala

**Affiliations:** Renal Division, Department of Health Sciences, ASST Santi Paolo e Carlo, University of Milan, Milan, Italy; Renal Division, Department of Health Sciences, ASST Santi Paolo e Carlo, University of Milan, Milan, Italy; UCL Department of Renal Medicine, Royal Free Hospital, London, UK

To the editor,

Multiple studies have emphasized the importance of genetic screening for kidney stone disease (KSD). Monogenic causes of KSD are estimated to be responsible for up to 15% of cases in specialized clinics [1]. Routine genetic testing for all stone formers remains impractical due to cost and resource constraints. Therefore, careful clinical selection remains essential to ensure cost-effective and meaningful genetic testing. As such, identifying predictive clinical features that indicate a higher likelihood of monogenic disease is critical for optimizing genetic testing strategies. Previous authors have provided evidence for the usefulness of clinical parameters in estimating pretest probability of a monogenic cause of nephrolithiasis, with current guidelines suggesting prioritizing patients with features such as early onset of disease, bilateral stones, nephrocalcinosis or a positive family history for further genetic evaluation [2, 3].

We retrospectively analysed all patients attending the metabolic kidney stone clinic at the Royal Free Hospital between September 2022 and September 2024 who underwent genetic testing to determine which clinical parameters were most predictive of a positive genetic diagnosis. We hypothesised that a scoring system combining certain clinical features (Table 1) might indicate a higher likelihood of a monogenic cause of kidney stones. This could then help determine which patients should have genetic testing.

**Table 1:** Scoring system devised by combining each selected parameter to produce a cumulative score.

Parameter	Score
Family history	
Positive family history in first-degree relative	2
Positive family history in second-degree relative	1
No family history	0
First stone episode (age in years)	
<18	4
18–30	2
>30	0
Presence of nephrocalcinosis	4
Recurrence	
>4 recurrences in the last 2 years	4
2–4 recurrences in the last 2 years	2
>2 recurrences in last ≥3 years	1
No recurrence	0
eGFR (ml/min/1.73 m^2^) at the time of test	
<30	4
30–60	2
60–90	1
>90	0

Family history is defined as a positive family history of kidney stones in at least one of the patient’s immediate relatives. Recurrence was defined as either spontaneous stone passage or need for a surgical procedure in the period indicated.

Patients were selected for genetic testing based on either clinical suspicion of a specific genetic disorder or failure of clinical and biochemical investigations to establish a definitive diagnosis. Patients underwent next-generation sequencing or whole genome sequencing with a focused exome analysis using the R256 nephrolithiasis and nephrocalcinosis gene panel, provided by the NHS Genomic Medicine Service [4].

Clinical data, including age at first kidney stone episode, family history, nephrocalcinosis presence, recurrence rate and estimated glomerular filtration rate (eGFR), were collected. A weighted cumulative risk score was applied, integrating established monogenic KSD risk factors. Patients with a confirmed genetic mutation (‘positive group’) were compared with those without a mutation (‘negative group’). Statistical analyses included *t*-tests to assess differences between groups, with significance defined as *P* < .05.

We found that 6 of the 35 patients tested (17%) had a confirmed monogenic disorder. Three clinical parameters were significantly associated with a positive genetic diagnosis showing an increased likelihood of having an underlying monogenic disorder, which were first stone episode before 18 years of age (*P* = .0107), presence of nephrocalcinosis (*P* = .0040) and a higher cumulative risk score (*P* = .0073).

Limitations of this study include its small sample size, single-centre design and retrospective selection of the cohort from patients already referred for testing. Although based on a small and highly selected sample of patients, this study confirmed that genetic testing is more likely to yield a positive outcome in patients who form stones at a young age and/or have nephrocalcinosis. Thus it supports the idea that clinicians should enquire about age at the first stone in all stone formers. However, it also highlighted that a higher cumulative risk score was associated with an increased risk of having a monogenic disorder. This is important, as the implementation of a weighted risk scoring system, which combines and weights multiple parameters, might be a pragmatic approach to enhance the precision and stratification possibility of genetic screening.

Although future multicentre studies with larger cohorts and prospective designs are necessary to validate these findings, we showed that an easily calculated clinical risk score can help to prioritise requests for genetic screening in a stone cohort. Until genetic screening of all stone formers is routine practice, a method of prioritisation over and above age at the first stone and presence of nephrocalcinosis is needed.

## References

[1] Howles SA, Thakker RV. Genetics of kidney stone disease. Nat Rev Urol 2020;17:407–21. 10.1038/s41585-020-0332-x 32533118

[2] Geraghty R, Lovegrove C, Howles S et al. Role of genetic testing in kidney stone disease: a narrative review. Curr Urol Rep 2024;25:311–23. 10.1007/s11934-024-01225-5 39096463 PMC11374836

[3] Ferraro PM, D’Ambrosio V, Gambaro G et al. A clinical screening algorithm for primary hyperoxaluria type 1 in adults on dialysis. Nephrol Dial Transplant 2023;39:367–70. 10.1093/ndt/gfad184 PMC10828199 37708050

[4] PanelApp . Nephrocalcinosis or nephrolithiasis (version 5.1). https://panelapp.genomicsengland.co.uk/panels/149/ (29 September 2025, date last accessed).

